# Interactions between endoplasmic reticulum stress and extracellular vesicles in multiple diseases

**DOI:** 10.3389/fimmu.2022.955419

**Published:** 2022-08-11

**Authors:** Jingyao Ye, Xuehong Liu

**Affiliations:** ^1^ Shandong University of Traditional Chinese Medicine, Jinan, China; ^2^ The Third School of Clinical Medicine of Guangzhou University of Chinese Medicine, Guangzhou, China

**Keywords:** endoplasmic reticulum stress, extracellular vesicles, interactions, multiple diseases, UPR signaling pathways

## Abstract

Immune responses can severely perturb endoplasmic reticulum (ER) function. As a protein-folding factory and dynamic calcium storage compartment, the ER plays a pivotal role in resisting pathogens and in the development of autoimmune diseases and various other diseases, including cancer, cardiovascular, neurological, orthopedic, and liver-related diseases, metabolic disorders, etc. In recent years, an increasing number of studies have shown that extracellular vesicles (EVs) play important roles in these conditions, suggesting that cells carry out some physiological functions through EVs. The formation of EVs is dependent on the ER. ER stress, as a state of protein imbalance, is both a cause and consequence of disease. ER stress promotes the transmission of pathological messages to EVs, which are delivered to target cells and lead to disease development. Moreover, EVs can transmit pathological messages to healthy cells, causing ER stress. This paper reviews the biological functions of EVs in disease, as well as the mechanisms underlying interactions between ER stress and EVs in multiple diseases. In addition, the prospects of these interactions for disease treatment are described.

## Introduction

**Figure 1:**
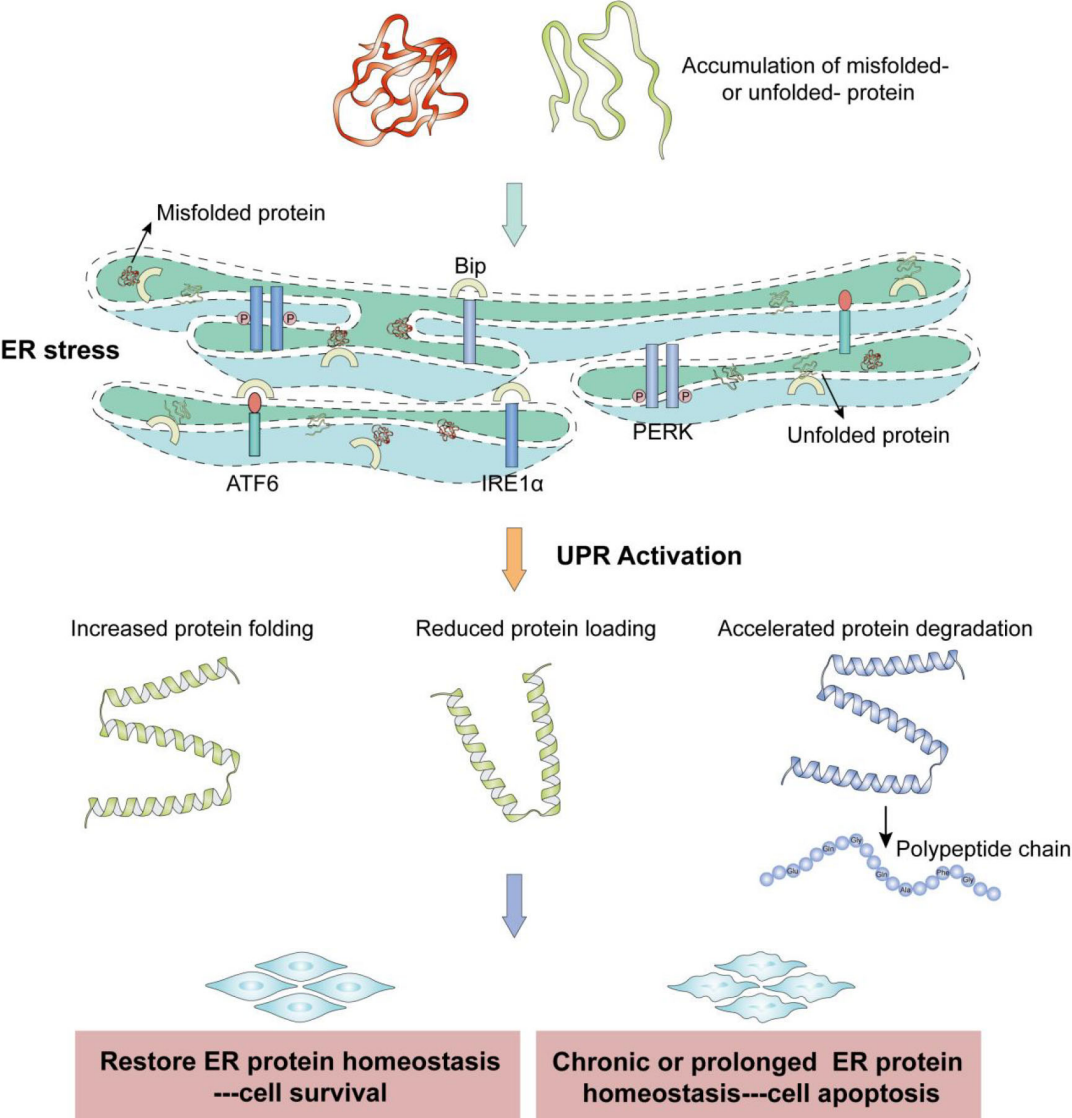
The unfolded protein response and pro-survival versus pro-death roles of endoplasmic reticulum stress. Accumulations of misfolded proteins and unfolded proteins cause ER stress. ER stress triggers the UPR, which is mediated by the three mammalian UPR transducers IRE1, PERK, and ATF6. Under conditions of ER stress, these proteins have various signal-mediated transcriptional effects that ameliorate ER stress and promote cell survival, but cell apoptosis occurs if ER protein homeostasis is not recovered.

In all eukaryotic cells, the endoplasmic reticulum (ER) is an extensive membranous labyrinth of branching tubules and flattened sacs. The ER is a protein-folding factory and dynamic calcium storage compartment in the cell. Approximately one third of all cellular proteins are modified post-translationally, with the folding and oligomerization of proteins occurring in the lumen of the ER. Moreover, the ER can continuously respond to environmental cues to release calcium (1). The ER is exquisitely sensitive to alterations in homeostasis and plays a key role in its maintenance, eventually resulting in adaptation for survival or induction of apoptosis through the action of signal transduction pathways. Early steps in the maturation of secretory proteins take place in the ER. If the influx of nascent proteins exceeds the folding capacity of the ER, or the abundance of unfolded polypeptides exceeds the ER’s processing capacity, an imbalance is created. This effect causes unfolded or misfolded proteins to accumulate in the ER lumen, perturbing the normal physiological state of the ER, which results in ER stress. Under ER stress, glucose-regulated protein 78 (GRP78) dissociates from the three transmembrane ER-stress mediators, which activates the unfolded protein response (UPR) signaling pathway. The ER can then return to its normal physiological state by altering the transcriptional and translational programs of the cell to cope with stressful conditions and resolve the protein-folding defect. The three transmembrane ER-stress mediators (also known as the main UPR signaling cascades) include IRE1α (inositol-requiring 1α), ATF6 (activating transcription factor 6), and PERK (pancreatic endoplasmic reticulum kinase). In response to ER stress, IRE1α is autophosphorylated, which results in a frameshift of XBP1 and translation of an XBP1 isoform with potent activity as a transcription factor (referred to here as active XBP1) (2, 3). In parallel, after release of binding immunoglobulin protein (BIP, an ER chaperone), ATF6 is cleaved by site-1 and -2 proteases in the Golgi apparatus. Cleaved ATF6 and the active XBP1 isoform mainly induce the transcription of genes encoding ER chaperones and enzymes to promote protein folding, maturation, secretion, and ER-associated protein degradation in parallel pathways (4, 5). After the release of BIP, PERK phosphorylates eIF2α to inhibit general protein translation, enabling dedicated translation of ATF4 to induce autophagy or activate apoptosis. Additionally, PERK can promote cell survival by preventing the influx of additional nascent polypeptides into an already saturated ER lumen (6). However, severe, protracted, or uncompensated ER stress can cause the UPR to fail, resulting in cell death, often by apoptosis (**Figure 1**).

Extracellular vesicles (EVs) are a heterogeneous group of cell-derived membranous structures that are naturally released by nearly all types of cells and are widely distributed in a variety of bodily fluids, including blood, urine, peritoneal fluid, synovial fluid, and breast milk. EVs can be broadly divided into two categories, ectosomes and exosomes (7). Ectosomes are derived from the plasma membrane and generated by its direct outward budding, producing large oncosomes (~1–10 μm), apoptotic bodies (~1–5 μm), microvesicles (MVs, ~200–1000 nm), and exomeres (<50 nm). In contrast, exosomes (~40–160 nm), which are derived from the endosomal pathway, are primarily released by fusion of the plasma membrane and multivesicular bodies (MVBs) (8). EVs can encase multiple types of bioactive molecules, including RNA (e.g., mRNA, miRNA, mtRNA, and lncRNA), lipids, metabolites, cytoplasm, and cell surface proteins. The presence of DNA in EVs is controversial. One study suggested that all cellular components may exist in EVs, whereas recent studies have demonstrated that double-stranded DNA (dsDNA) and DNA-binding histones are not carried by EVs (9). These conflicting findings indicate that more studies exploring the composition of EVs are needed. To date, knowledge regarding the transport of lipid mediators by EVs is fragmented. Some recent studies have analyzed resolvins, prostaglandins, and polyunsaturated fatty acids (PUFAs) in EVs (10, 11). Nevertheless, the composition and function of lipids in EVs and the ER remain unclear, which may be due to the physiological function of the ER as a protein-folding factory. EV cargo is sheltered from being degraded by extracellular enzymes present in biological fluids, thus maintaining biological stability over comparatively long periods of time. For this reason, EV cargo, packaged within relatively stable membrane-bound structures, has been detected in almost all bodily fluids and circulates systemically. Different dimensions and surface molecules are hypothesized to affect the ability of EVs to recognize and capture recipient cells (12). EVs are vital information carriers that transfer their cargo from donor to recipient cells through protein interactions, endocytosis, or direct membrane fusion. However, the mechanisms of EV uptake and cargo delivery remain incompletely characterized (13). One study demonstrated that the phenomenon is highly specific and possibly depends on both the phenotype of the EV subtype and the extracellular characteristics of the acceptor cells (14). In contrast, another study reported that EV-cell interactions are unspecific and stochastic (15). We speculate that these mechanisms are influenced by multiple factors, such as the physiological or pathological state of the donor cell, the stimuli that modulate their production and release, and the molecular mechanisms modulating the physiological or pathological processes in recipient cells (16). It seems likely that EV uptake involves more than one mechanism, with multiple pathways working together simultaneously to modulate this process. Currently, several methods are employed to isolate EVs, including ultracentrifugation, polymer precipitation, size-based isolation techniques, with each method demonstrating advantages and disadvantages.

The formation of EVs is dependent on the endosomal sorting complex required for transport (ESCRT) or protein complexes of saturated lipid ceramides. The molecular machineries that act at different steps of EV biogenesis, including ESCRT proteins and generation of ceramide by sphingomyelinase, are common to exosomes and microvesicles (17–19). Both pathways can be enhanced by ER stress mediated by IRE1α and PERK to promote the release of EVs (20–22). Thus, ER stress enhances the ability of EVs to interact with cells and promotes the development of disease, suggesting that ER stress-dependent EVs may provide suitable biomarkers for early disease diagnosis. This paper reviews and summarizes the interactions between ER stress and EVs in multiple diseases and their influence on disease development (**Tables 1**, **2**), highlighting potential new targets for disease treatment.

**Table 1:** The role of ER stress and EVs in different diseases (miRNA/CircRNA).

Donor cells	Content	Recipient cells	Biological function	Targets	Reference
HNSCC,Cancer cells	MiRNA-424-5p	VECs	Inhibit the angiogenesis and migration of VECs	LAMC1/Wnt/β-catenin	(23)
BC,Cancer cells	MiRNA-27a-3p	Mφs	Promote tumor immune evasion	PTEN/AKT/PI3K/PD-L1	(24)
	Circ_0001142	Mφs	Promot the growth and metastasis of breast cancer	MiRNA-361-3p/PIK3CB	(25)
OSCC,Cancer cells	MiRNA-181a-3p	muscle cells	Activate the atrophy and apoptosis of muscle cells	TERS	(26)
GC,HER2-positive GC cells	MiRNA-301a-3p	trastuzumab sensitive cells	Spread trastuzumab resistance	LRIG1/IGF-1R/FGFR1	(27)
HCC,cancer cells	MiRNANA-23a-3p	Mφs,T cells	Induce Mφs to trend toward TAMs,Promot immune escape	PI3K/AKT,PD-L1	(28)
acute alcoholic liver injury,serum	MiRNANA-122	hepatocytes	Not mentioned	eIF2α,IRE1α	(29)
brittle diabetes,MSCs	MiRNANA-21	β-cell	Increase β-cell viability and inhibite β-cell apoptosis under hypoxic conditions and promoted islet survival and function	ER stress and p38/MAPK	(30)
AD, Serum	CircAXL/MiRNA-1306-5p	neuronal cells	Attenuate Aβ1-42-induced neurotoxicity	PDE4A	(31)
CNSD,H/R-HUVECs	MiRNA-199a-5p	neuronal cells	Inhibit BIP and apoptosis and inflammation associated with ER stress	ER stress	(32)
SCI,BMSCs	MiRNA-9-5p	PC12 cells	Alleviate apoptosis, inflammation and ER stress	HDAC5/FGF2	(33)
IVDD,MSC	Not mentioned	NPCs	Attenuate the rate of apoptosis in human NP cells	AKT/ERK	(34)
	MiRNA-31-5p	EPCs	Protect EPCs from oxidative stress-induced apoptosis, and inhibit oxidative stress-induced calcification	ATF6	(35)
Renal I/R injury,BMSCs	MiRNA-199a-5p	Renal tubular epithelial cells	Slow down cell death and apoptosis and effectively protect the kidney	BIP	(36)

**Table 2:** The role of ER stress and EVs in different diseases (proteins and others).

Donor cells	Content	Recipient cells	Biological function	Targets	Reference
OSCC,Cancer cells	PD-L1	Mφs	Drive tumor development and promote tumor immune evasion	PD-L1/M2 *Mφs*	(37)
NPC,cancer cells	ERp44	adjacent cells	Promot cell proliferation and migration, reducing cisplatin sensitivity	NF-κB	(38)
Cancer,Cancer cells	Minpp1 isoform -2	Not mention	Help ECM remodeling promote tumor cell growth	InsPs	(39)
VC,vascular smooth muscle cells	Grp78	VSMCs	Promote the formation of calcium phosphate crystals	PERK-ATF4	(40)
Hepatic steatosis,adipocyte	Resistin	hepatocytes	Lead to ER stress and hepatic steatosis	Resistin/AMPKα	(41)
Hepatic steatosis,adipocyte	Akr1b7	hepatocytes	Trigger hepatic steatosis and exacerbates NASH and elevating glycerol levels	Not mentioned	(42)
T1DM,β-cell	GAD65,IA-2insulin/insulinogen	T cells	Promot antigen presentation and T-cell activation, promoting β-cell autoimmunity	Not mentioned	(43)
DPN,Schwann Cells	IRE1α,GRP78	DRGn	Induce ER stress	Bcl-2,Bax	(44)
T3cDM, cancer cells	AM	β-cell	Induct failure of the UPR,increased β-cell dysfunction and death	ER stress	(45)
IVDD,USCs	Not mentioned	NPCs	Inhibit ER stress-induced apoptosis of NPCs	AKT/ERK	(46)
ONFH,PRP	Growth factors	ECs and osteoblasts	Promot angiogenesis,maintain the osteogenic capacity of osteoblasts	AKT/ERK,VEGF-A	(47)
Endothelial corneal dystrophy,MSC	Not mentioned	HCECs	Reduced apoptosis of HCECs	AKT	(48)

CNSD, central nervous system disease; SCI, spinal cord injury; HNSCC, head and neck squamous cell carcinoma; HUVECs, human umbilical vein endothelial cells; OSCC, oral squamous cell carcinoma; BC, breast cancer; GC, gastric cancer; HER2, human epidermal growth factor receptor 2; NPC, nasopharyngeal carcinoma; HCC, hepatocellular carcinoma; MSC, mesenchymal stem cell; DPN, diabetic peripheral neuropathy; T3cDM, type 3c diabetes mellitus; AD, Alzheimer’s disease; H/R, hypoxia/reoxygenation; BMSC, bone marrow mesenchymal stem cell; IVDD, intervertebral disc degeneration; USC, urine-derived stem cell; ONFH, osteonecrosis of the femoral head; PRP, platelet-rich plasma; I/R, ischemia/reperfusion; VC, vascular calcification; HCEC, human corneal endothelial cell; EC, endothelial cell; EPC, endplate chondrocytes;DRGn, dorsal root ganglion neurons; AM, adrenomedullin.

## Cancer

ER stress is involved in the occurrence, development, and metastasis of tumors induced by EVs. In addition to affecting tumor cells themselves, ER stress has corresponding effects on surrounding cells through intercellular effects mediated by EVs. Patients with head and neck squamous cell carcinoma (HNSCC), like most head and neck tumors, are profoundly jeopardized by recurrence or metastasis (49), which can occur by HNSCC cells directly entering the blood circulation system. Therefore, exploring the impacts of HNSCC cells on intratumoral vessels is crucial. Research has shown that ER stress not only directly regulates vascular endothelial cells (VECs), but also induces HNSCC cells to release mir-424-5P contained in EVs, which inhibits the expression of laminin gamma 1 (LAMC1) (23), a protein essential for the assembly of basement membranes as well as tumor angiogenesis and development. Furthermore, miR-424-5p repressed the LAMC1-mediated Wnt/β-catenin signaling pathway, thus inhibiting angiogenesis, migration of human umbilical vein endothelial cells (HUVECs), and tumor development. This finding provided new insight into the complex mechanism of anti-angiogenesis in HUVECs.

Oral squamous cell carcinoma (OSCC), another cancer of the head and neck area, is associated with low survival rates due to metastasis or local uncontrollable recurrence (50). Programmed cell death-ligand 1 (PD-L1) inhibits anti-tumor immunity and promotes tumor immune escape by establishing an immunosuppressive microenvironment, whereas M2 macrophages (Mφs) are known to infiltrate the tumor microenvironment (TME) and promote tumor progression by damaging the immune response of cytotoxic CD8^+^ T cells. One study reported that ER stress-related proteins were highly expressed in OSCC specimens, with abundant CD163^+^ Mφs and high PD-L1 expression in the tumor stroma (37). In addition, ER stress promoted the release of PD-L1-rich EVs from OSCC cells. Subsequently, these EVs transferred PD-L1 and ER stress to Mφs, thus upregulating PD-L1 expression to promote M2 polarization. M2 Mφs drive tumor development and PD-L1 contributes to tumor immune evasion; thus, their expression levels were negatively correlated with the overall survival of patients. These results suggest that the ER stress-OSCC-EV-Mφs axis promotes tumor development through immune mechanisms, which may provide a new direction for the future treatment of OSCC.

Notably, patients with OSCC are more likely to develop progressive muscle atrophy, a hallmark of cancer cachexia. ER stress, which is prevalent in invasive cancers, can be transferred to neighboring or distant cells through intercellular signaling *via* EVs, known as transmissible ER stress (TERS). However, the specific mechanism of action in muscle remodeling (such as in cachexia) remains unclear. In OSCC, ER signaling was shown to occur through EVs, which were rich in mir-181a-3p (26). mir-181a-3p activated muscle atrophy and apoptosis pathways through TERS signaling in muscle cells, resulting in cachexia. Therefore, mir-181a-3p may provide a candidate target for alleviating muscle atrophy in patients with cancer cachexia, confined not only to OSCC but also cachexia associated with other tumors. However, whether the effects of mir-181a-3p occur through autophagy, sensitizing muscle cells to apoptosis signals, or other pathways requires further study.

In addition to OSCC, breast cancer (BC) development is also promoted by ER stress and EVs through immune escape. As in as OSCC, research has demonstrated that PD-L1-enriched EVs derived from BC cells are transferred to Mφs and PD-L1 expression is upregulated through several pathways (24). The expression of mir-27a-3p was increased in EVs under ER stress, targeting the scaffold protein membrane-associated guanylate kinase inverted 2 (MAGI2), which is required for the negative regulation of phosphatase and tensin homolog (PTEN). Reduced MAGI2 expression downregulated the tumor suppressor PTEN and activated the phosphatidyl inositol 3-kinase (PI3K)/AKT signaling pathway, which upregulated PD-L1 in Mφs and promoted immune escape of BC cells. In other words, mir-27a-3p in BC-derived EVs induced by ER stress upregulated the expression of PD-L1 in Mφs through the PTEN/AKT/PI3K axis, causing immune escape. This finding may provide a new anti-tumor immunotherapy target by activating the patient’s own immune system. It follows that PD-L1 delivered by EVs provides an important target for inhibiting tumor immune escape. In addition to mir-27a-3p, circ_0001142 in EVs was shown to enter tumor-associated Mφs under ER stress, interfere with Mφs autophagy, and induce M2 polarization through the mir-361-3p/PIK3 catalytic subunit beta pathway, thus promoting BC growth and metastasis (25). These findings suggest that the contents of EVs under ER stress have multiple rather than single targets, and the complex pathogenesis may require simultaneous inhibition of multiple pathways.

Although chemotherapy is a common treatment for BC, chemotherapy drug resistance is one of the main reasons for poor prognosis. Combined treatment with paclitaxel and JI017, a new herbal medicine containing extracts from *Angelica gigas*, *Zingiber officinale* Roscoe, and *Aconitum carmichaeli*, reduced cell survival and increased cell death in paclitaxel-resistant and typical BC cells, while increasing ER stress (51). Excessive and prolonged ER stress is known to sensitize chemoresistant cancer cells and mediate apoptosis. JI017 induced ER stress in BC cells by releasing GRP78 from EVs. Additionally, JI017 induced increased production of intracellular reactive oxygen species (ROS) and nicotinamide dinucleotide phosphate (NADPH) oxidase (NOX4) activity, which inhibited the sarcoendoplasmic reticulum Ca^2+^-ATPase (SERCA) pump. Thus, JI017 induced ER stress and cell death in BC cells, overcoming paclitaxel resistance by blocking the epithelial-mesenchymal transition. Together, these findings suggest that information regarding ER stress can be transmitted through EVs to influence the disease process, and, in turn, EVs can induce ER stress and influence disease.

GRP78 plays a key role in tumor proliferation, angiogenesis, and immune resistance, as well as in antitumor drug resistance, and its overexpression has been detected in various cancers, including colon cancer. GRP78 is found in the ER, plasma membrane, and cytoplasm of tumor cells, but the mechanism by which GRP78 is secreted remains unclear. GRP78 is reportedly secreted from colon cancer cells through exosomes. In one study, histone deacetylase (HDAC) inhibitors were used to block HDAC6 activity, which led to increased GRP78 acetylation. The acetylated GRP78 bound to another kinase, thus preventing GRP78 from sorting into MVBs and ultimately inducing GRP78 aggregation in the ER. This prevented the release of GRP78 from EVs and promoted kinase-mediated autophagy, which impaired tumor growth (52). In addition, this study demonstrated the role of EVs in expelling intracellular waste, as was previously hypothesized.

EVs are not the only way to activate ER stress, which may provide a new approach to anti-tumor drug resistance that warrants further study. Drug resistance in BC, gastric cancer (GC), and nasopharyngeal carcinoma (NPC) is related to EVs and ER stress. The standard first-line therapy, chemotherapy drugs combined with trastuzumab, is not effective for some patients with human epidermal growth factor receptor 2 (HER2) positive GC. mir-301a-3p in EVs induced by ER stress was shown to downregulate expression levels of leucine-rich repeats and immunoglobulin-like domains protein 1 (LRIG1) by directly binding to the 3′-UTR of LRIG1 mRNA (27). Subsequently, the expression of insulin like growth factor 1 receptor (IGF-1R) and fibroblast growth factor receptor 1 (FGFR1) was promoted, mediating trastuzumab resistance. In addition, mir-301a-3p could be transmitted from trastuzumab-resistant to trastuzumab-sensitive cells through EVs, spreading trastuzumab resistance. This finding may provide a new target for anti-tumor drug resistance. In addition to trastuzumab resistance, cisplatin resistance can result from ER stress through EVs. ER resident protein 44 (Erp44) is a member of the protein disulfide isomerase family that is activated by the UPR during ER stress. Erp44 plays an important role in tumor development, not only in promoting cell proliferation and migration, but also in reducing cisplatin sensitivity by activating nuclear factor kappa B (NF-κB) to inhibit cell apoptosis. Under ER stress, NPC cells were shown to release many Erp44-rich EVs and transmit ER stress signals through EVs (38). For example, decreased Erp44 levels in EVs inhibited the proliferation of CNE2 cells (poorly differentiated NPC cells) and increased cisplatin sensitivity. Thus, under ER stress, tumor cells produced EVs containing Erp44, which migrated to neighboring cells, enhanced chemotherapeutic resistance, and promoted the proliferation of CNE2 cells. These results provided new insight into the role of EVs in tumor drug resistance.

Taken together, these studies suggest that the cause and spread of resistance is related to both the ER and EVs, but resistance to different drugs may be caused by different pathways, necessitating different treatments. The mechanism by which cancer cells penetrate the endothelium, a key step in cancer metastasis, remains unknown. Endothelial tight junction (TJ) proteins, including zonula occludens-1 (ZO-1) and claudin-5 (CLDN5), control the permeability of endothelial monolayers and play an important role in vascular integrity. Downregulation or loss of TJs leads to increased endothelial permeability and promotes cancer progression. In one study, intravenous injection of EVs from human cervical squamous carcinoma HeLa cells (HeLa-EVs) triggered ER stress in endothelial cells, reduced the expression levels of ZO-1 and CLDN5 in blood vessels, and increased the vascular permeability of vital organs, significantly increasing tumor metastasis (53). However, inhibition of ER stress prevented the downregulation of ZO-1 and CLDN-5 by HeLa-EVs. These results indicated that HeLa-EVs damaged vascular integrity *via* downregulation of TJ-related proteins like ZO-1 and CLDN5 in endothelial cells, thus activating ER stress and promoting tumor metastasis. In short, EVs secreted by some tumor cells can trigger ER stress in endothelial cells and destroy their integrity, thus allowing tumor cells to penetrate vascular walls to achieve tumor metastasis. Interestingly, inhibition of two highly expressed miRNAs (miR-1290 and miR-3960) in EVs did not reverse the inhibitory effect of HeLa-EVs on CLDN5 and ZO-1 expression, suggesting the effect of EVs may be miRNA-independent. Additional studies are needed to prove this hypothesis. In HNSCC, ER stress inhibits tumor angiogenesis through EVs, whereas EVs promote angiogenesis in HeLa cells by triggering ER stress. These opposing effects suggest that the mutual actions of ER stress and EVs on disease development are not necessarily inhibition or promotion, but may be related to changes in the microenvironment. Moreover, the roles of ER stress and EVs in one disease are not necessarily applicable to another, which complicates treatment.

Inositol polyphosphates (InsPs) are natural signaling molecules that maintain cellular calcium homeostasis. Among InsPs, myo-inositol-1,2,3,4,5,6-hexaphosphate (InsP6) and its derivatives are anti-proliferative agents that counteract tumorigenic processes, thus reverting a cell to normalcy. Research has revealed that multiple inositol polyphosphate phosphatase1 (Minpp1) removes the 3-phosphate from higher InsPs (such as InsP6) to convert them to lower InsPs (such as InsP1), repressing the anti-proliferative effect (39). Normally, low EV-associated protein expression is a defense mechanism by which cells respond to ER stress by inducing the expression of specific genes to reestablish a stable environment. During ER stress, relatively small EVs (~95 nm) are secreted and transmitted from cells in an uninhibited manner, accompanied by increased expression of the EV-related Minpp1 isoform-2 enzyme, which is almost dormant under normal conditions. Minpp1 isoform-2 is speculated to contribute to tumor cell proliferation by protecting tumor cells from the anti-proliferative effects of extracellular IP6 or by promoting extracellular matrix (ECM) remodeling. Minpp1 isoform-2 may also promote tumor cell growth by activating adjacent protein cassettes that prevent cell death and influence the composition of the TME. These findings suggest that overexpression of Minpp1 isoform-2 in ER stress induced-EVs may be a cellular stress alert. Whether this occurs in all tumors or only in specific diseases remains unclear. Additionally, whether Minpp1 isoform-2 is an effective target to inhibit tumor progression or a promising biofluid-based, non-invasive, early cancer biomarker remains to be determined.

Under ER stress, tumor cells are stimulated to secrete EVs rich in certain tumorigenic molecules, which are transferred to normal cells and play a carcinogenic role. Additionally, the function of the ER can be affected by EVs, thus affecting disease development. For example, EVs may directly transport large amounts of non-specific cargo to the ER, such as mRNA transcripts or misfolded proteins, thus inhibiting its function and inducing ER stress. Alternatively, EVs derived from tumor cells (T-EVs) can deliver specific molecules that influence ER stress pathways. For example, long-term exposure of SV-HUC cells (SV40-transformed HUC, a human urothelial cell line) to bladder cancer T-EVs was shown to stimulate the UPR in the ER (54). Long-term induction of UPR signals activated IRE1α, the survival branch of the UPR signaling pathway, which increased expression levels of NF-κB and the inflammatory cytokine leptin. Additionally, long-term induction of UPR signals downregulated pro-apoptotic protein C/EBP homologous protein (CHOP), which activated the DNA damage response and ultimately led to malignant transformation. Moreover, inhibition of ER stress prevented T-EV-induced transformation. Therefore, protein upregulation driven by T-EVs may be a new mechanism of tumorigenesis. In other words, T-EVs promote malignant transformation of susceptible cells by inhibiting pro-apoptotic signals and promoting ER stress-induced UPR and inflammation in tumors. This effect may underlie the role of bladder cancer T-EVs in the carcinogenesis of adjacent urothelial regions and induction of urothelial carcinoma in the urinary bladder. Therefore, inhibition of EV internalization and ER stress in urethral epithelial cells may prevent malignant transformation, providing insights into the underlying mechanism of tumor recurrence.

In addition, T-EVs can be delivered not only to normal non-tumorigenic cells and Mφs, but to other immune cells, such as T lymphocytes. P38 mitogen-activated protein kinase (MAPK) plays a key role in the pro-apoptotic effect of ER stress. Pancreatic cancer cells were shown to secrete EVs that are absorbed by T lymphocytes, inducing phosphorylation of P38 MAPK by upregulating JUN expression, and transmitting ER-derived signals to the nucleus through the PERK-eIF2α-ATF4-CHOP signaling pathway (55). In this manner, cells can sense ER stress, leading to T cell apoptosis and subsequent immunosuppression. Interestingly, changes in expression levels of dual-specificity phosphatase 1 (DUSP1), a protein phosphatase that negatively regulates the MAPK pathway, were consistent with changes in p38 phosphorylation, which may serve as an important negative feedback control mechanism to reduce p38 MAPK overactivity. The strong stimulating effect of EVs on P38 MAPK activity exceeded the regulation of DUSP1 feedback activity, indicating the powerful function of EVs. In short, EVs from pancreatic cancer cells were transferred to peripheral T lymphocytes and altered their gene expression profile, reducing their anti-tumor ability and promoting tumor development through the apoptosis of immune cells. Notably, pancreatic cancer often leads to diabetes and metabolic disorders. Interactions between ER stress and EVs in pancreatic cancer affect not only T cells, but also B cells, which will be discussed in the chapter on metabolism-related diseases.

In summary, EVs can both inhibit and promote tumor development under ER stress, and both inhibit, cause, and spread drug resistance. We speculate that these phenomena may be closely related to the TME. Thus, manipulating EVs under ER stress, enhancing ER stress in tumor cells, and regulating the TME to suppress disease development provide directions for future research.

## Diseases of the heart and lungs

In addition to the above-mentioned cancers, interactions between ER stress and EVs have an impact on the development of lung cancer, which will be discussed in this section. PPZ023 (1-(2-(ethylthio)benzyl)-4-(2-methoxyphenyl)piperazine), a novel peroxisome proliferator-activated receptor gamma (PPARγ) ligand candidate, has a powerful anticancer effect against tumor growth. One study reported that PPZ023 reduced the viability of non-small cell lung cancer (NSCLC) cells, increased lactate dehydrogenase (LDH) cytotoxicity, enhanced caspase-3 activity, induced cell death by producing ROS (apoptotic cells with impaired ER function are more likely to produce ROS), and triggered the release of mitochondrial cytochrome c (56). In addition, PPZ023 treatment activated PERK and phosphorylation of eIF2α, leading to upregulation of CHOP and ultimately the death of NSCLC and radio-resistant NSCLC cells through the transport of ER stress-induced EVs. This finding suggests that ER stress can be delivered to tumor cells through EVs, thus promoting apoptosis and disease suppression, which may be applicable to many diseases.

Interactions between ER stress and EVs are not limited to cancer in the lungs. Some forms of chronic lung inflammation, such as silicosis caused by the inhalation of silica dust (SiO_2_), are closely related to both. Exposure to silica dust significantly increased the abundance of EVs secreted by Mφs, and SiO_2_-induced Mφs-EVs effectively promoted the differentiation, proliferation, and migration of myofibroblasts (57). Meanwhile, treatment with EV inhibitors inhibited silica-induced pulmonary fibrosis and inflammation. Further, treatment with SiO_2_-induced Mφs-EVs upregulated expression levels of type I collagen, α-smooth muscle actin (SMA), BIP, XBP1s, and P-EIF2α. Bronchoalveolar lavage fluid showed reduced pulmonary fibrosis and expression of tumor necrosis factor (TNF)-α, interleukin (IL)-1β, and IL-6 after treatment with EVs inhibitors. These results indicated that the fibrogenic effect of SiO_2_-induced Mφs-EVs depended on ER stress. Indeed, treatment with the ER stress inhibitor 4-phenylbutyric acid (PBA) reversed this effect. In short, inhibition of ER stress reversed the fibrotic phenotype of activated myofibroblasts. Inhibition of EV production also inhibited silica-induced pulmonary fibrosis and inflammation in mice. This study demonstrated that fibroblast activation after SiO_2_-induced Mφs-EV treatment was dependent on ER stress activation. However, the identity of the molecular or cellular cargo inside EVs remained unclear. In addition, the mechanism by which ER stress is activated by SiO_2_-induced Mφs-EVs warrants further study. Nevertheless, these results suggest that Mφs can act as both receivers and senders of ER stress mediated by EVs. In mice with ventilator-induced lung injury (VILI), inhibition of ER stress reduced the release of EVs, expression levels of PERK and Toll-like receptor 4 (TLR4), and lung injury (58). Although these results further supported that EV release is related to ER stress, determining the specific mechanism of action is a direction for future research. In another study, circulating EVs from mice with LPS-induced acute respiratory distress syndrome (ARDS) were injected into the tail veins of normal mice (59), resulting in reduced β-catenin and VE-cadherin expression in lung tissues. These results indicated that EVs caused damage to the alveolar capillary barrier. Further, expression levels of ER stress-related proteins GRP78 and CHOP were significantly upregulated in lung tissues, indicating that ER stress was activated, at least in part, by promoting endothelial dysfunction in mice receiving EVs from ARDS mice. However, the exact pathway and downstream mechanism of ER stress warrants further study.

EVs act on the ER to inhibit ER stress in certain situations, which can slow the progression of disease. For example, percutaneous coronary intervention after acute myocardial infarction and subsequent reperfusion may induce further cardiac injury in a process known as myocardial ischemia/reperfusion (MI/R) injury. In one study, a cellular MI/R injury model constructed using hypoxia/reoxygenation (H/R) induced severe ER stress, resulting in increased expression levels of GRP78, CHOP, IRE1α, and ATF6, which inhibited the PI3K/AKT pathway and promoted cell apoptosis (60). After treatment with EVs derived from human umbilical cord mesenchymal stromal cells (HuMSC-EVs), the harmful effects of H/R in cells were reduced, ER stress was inhibited, and survival of cells was increased. However, when treated with a PI3K/AKT inhibitor, the protective effect of HuMSC-EVs was partially reduced and expression levels of GRP78, CHOP, IRE1α, ATF6, and lytic caspase 3 were increased. HuMSC-EVs were speculated to improve ER stress-induced apoptosis by activating the PI3K/AKT signaling pathway. This finding may provide a new direction for the clinical treatment of MI/R injury.

Almost all patients with cardiovascular disease have some degree of vascular calcification (VC). VC increases the incidence of cardiovascular disease as well as mortality, and vascular smooth muscle cells mediate VC through various mechanisms. Inducing ER stress has been shown to increase the release of GRP78-loaded EVs from vascular smooth muscle cells, whereas GRP78 promotes the formation of calcium phosphate crystals in the collagen matrix, which promotes calcification (40). Activation of ER stress by favalin increased GRP78-loaded EV release and promoted calcification *via* the PERK-ATF4 pathway. None of these pathways were associated with apoptosis. In addition to GRP78, GRP94 expression has also been found to be increased in EVs, but has not yet been shown to promote calcification. Several drugs protect cardiomyocytes through the attenuation of oxidative and ER stress. Ticagrelor, which is widely used for cardioprotection, has anti-platelet as well as angiogenic effects. One study reported that cardiomyocyte-derived EVs pretreated with ticagrelor significantly reduced hyperglycemia-induced abnormal ROS production, prevented the development of apoptosis and ER stress, attenuated oxidative stress-related miRNA expression profiles, and enhanced endothelial cell migration and tube formation, thus exerting antioxidant and cardioprotective effects on hyperglycemic cardiomyocytes (61). These experiments demonstrated that EVs do not always induce ER stress and promote disease but may also inhibit ER stress and suppress disease. Therefore, further research should focus on inhibiting the disease promoting effects of EVs and promoting the disease inhibiting effects of EVs for therapeutic benefit.

## Liver-related diseases

Liver lipids play an important role in systemic metabolism, and excessive accumulation of lipids leads to liver steatosis, which can further develop into nonalcoholic steatohepatitis (NASH). One study demonstrated that plasma free fatty acids induced the inflammatory response (meta-inflammation) in hepatocytes through ER stress, thus overactivating the UPR while activating PERK signaling and the CHOP transcription factor in association with cyclooxygenase 2 expression (62). In addition, the PERK pathway and UPR were activated in hepatocytes co-cultured with EVs isolated from the supernatant of LPS-stimulated Mφs, resulting in meta-inflammation due to increased ROS production and related ER stress. Eventually, cholesterol protein targets were altered, which contributed to the development of obesity, fatty liver disease, and dyslipidemias. These results demonstrated that PERK signaling is a key mechanism in the protection of hepatocytes from protein deposition. Although the components of EVs that cause meta-inflammation are currently unknown, targeting PERK signaling is a clear direction for drug development. In addition to Mφs-derived EVs, adipocyte-derived EVs (A-EVs) can also cause hepatic steatosis. Resistin is a key cytokine that induces ER stress and leads to hepatic steatosis by inhibiting phosphorylation of 5′-adenosine monophosphate-activated protein kinase α (pAMPKα) on Thr172. Meanwhile, melatonin protects the liver through various mechanisms and inhibits the proliferation and invasion of hepatocellular carcinoma (HCC). One study demonstrated that A-EVs were rich in resistin and transported from adipose tissue to the liver (41). Resistin led to ER stress by inhibiting AMPKα signaling, which ultimately led to hepatic steatosis. However, treatment with melatonin reduced the production of resistin by inhibiting Bmal1 transcription and enhancing m6A RNA demethylation to degrade resistin mRNA. In short, melatonin reduced the flow of extracellular resistin from adipocytes to hepatocytes, which further alleviated hepatic steatosis induced by ER stress.

In addition to resistin from A-EVs causing liver steatosis through ER stress, ER stress-induced A-EVs can induce NASH. ER stress alters the proteome of EVs secreted by fat cells that coordinate lipid dynamics in the liver. Aldo-keto-reductase 1b7 (Akr1b7), a key regulatory factor in NASH induced by high fat (HFD) and methionine and choline-deficient (MCD) diets, was shown to induce hepatic steatosis by increasing glycerol levels and lipid accumulation in the liver (42). More importantly, ER stress-induced A-EVs triggered hepatic steatosis and exacerbated NASH by delivering extracellular Akrt1b7 to the hepatocyte cytosol and elevating glycerol levels. In contrast, Akr1b7 deficiency actively protected mouse livers from HFD and MCD-induced NASH. Because of the diversity of components in EVs, Akr1b7 may not be the only explanation for the effects of A-EV treatment on hepatic steatosis in mice. However, A-EVs may provide a potential therapeutic strategy to prevent or control obesity-induced NASH. Moreover, these results demonstrated the vicious cycle in which the EV and ER stress interact to promote disease progression. In a dietary mouse model of NASH, the lipotoxic ER stress response was activated and the expression of nucleus signaling 1 protein (ERN1, also named IRE1α) was increased (63), both of which are transducers of activated UPR in the livers of patients with NASH. The ribonuclease of IRE1α cleaved XBP1 mRNA to produce the spliced-variant XBP1s mRNA that coded for the XBP1 protein, which promoted the transcription of serine palmitoyltransferase genes. This transcription resulted in ceramide biosynthesis and stimulated the release of ceramide-rich EVs from liver cells. After being injected intravenously with IRE1α-stimulated hepatocellular-derived EVs, mice with diet-induced steatohepatitis exhibited increased accumulation of monocyte-derived Mφs in the liver. The proinflammatory EVs transmitted hepatocellular ER stress to Mφs, leading to inflammation and damage. Destruction or inhibition of IRE1α in hepatocytes reduced enterovirus release, liver injury, inflammation, and Mφs accumulation in mice fed a high-fat, high-fructose, and high-cholesterol diet. Although the study did not indicate which subtype of Mφs accumulated in the liver, the pro-inflammatory M2 subtype was likely observed. In short, ER stress was transmitted to Mφs *via* EVs in a ceramide-dependent manner, leading to development of NASH, which may be applicable to the development of other diseases.

In addition to NASH, ER stress and EVs also play pivotal roles in the development of alcoholic fatty liver disease (AFLD). Cytochrome P450 (CYP), a superfamily of enzymes, is involved in the metabolism of endogenous and exogenous compounds. For example, ethanol-inducible CYP2E1-mediated substrate metabolism leads to the production of ROS and active metabolites, with subsequent cytotoxic effects. One study reported increased CYP2E1 levels and ROS production in primary hepatocytes exposed to ethanol (64). Likewise, the abundance and protein content of circulating EVs in plasma increased, but their size did not change. Increased CYP2E1 levels and ROS production promoted ER stress, leading to the accumulation of nitrated and unfolded proteins. This effect caused damaged liver cells to release EVs rich in modified or unfolded CYP2E1 (and other CYP proteins), which interacted with target or neighboring cells to promote cell death by activating apoptotic signaling pathways. Similarly, ER stress activators increased EV secretion and CYP2E1 expression in hepatocytes. In contrast, EVs and CYP2E1 expression mediated by ethanol were significantly reduced after treatment with CYP2E1 inhibitors, powerful antioxidants, ER stress inhibitors, or EV secretion inhibitors. These results confirmed that nitrified CYP2E1 and other proteins in EVs may be secreted during CYP2E1-dependent oxidation and ER stress after ethanol exposure. Interestingly, PUFAs also inhibited CYP2E1 expression in EVs. This study provided insight into whether CYP2E1 can be used as a potential biomarker for alcohol exposure or AFLD. Alcohol can not only affect proteins in EVs such as CYP2E1, but can also affect miRNA, such as miRNA-122. In another study, levels of phosphorylated eIF2α, IRE1α, and ER-related proteins were significantly increased in the livers of alcohol-treated mice (29), suggesting that ER stress is activated in the pathogenesis of ethanol-induced acute alcohol injury. As an inhibitor of ER stress, PBA does not interfere with alcohol metabolism, but pretreatment with PBA reduced miR-122 expression in mouse EVs and significantly inhibited the transcription of ER stress-related proteins during the pathogenesis of ethanol-induced acute liver injury. These findings suggest that PBA may be an effective drug for acute alcoholic liver injury, but the specific mechanism of action of ER stress on miR-122 from EVs warrants further exploration.

Without intervention, alcohol-induced liver injury may progress to liver fibrosis. The above findings demonstrate that the etiology of disease is often related to multiple pathways. Thus, optimal results are often not achieved by intervening in a single pathway. Angiotensin I (Ang II) signaling plays an important role in the pathogenesis of fibrosis. Using an *in vitro* model of hepatic fibrosis in LX-2 cells, researchers reported that treatment with Ang II increased hematopoietic stem cell (HSC) activity, inflammation, ER stress, ROS production, and expression of ECM-related proteins (α-SMA, Col I, and Col III) (65). Ang II treatment also increased the expression of apoptosis signal-regulating kinase 1 (ASK1), which can mediate ER stress and lead to EV release from Ang II-activated HSCs. ASK1 inhibitors significantly reversed Ang II-induced liver fibrosis and ER stress. However, the specific mechanism of EVs downstream remains to be determined.

In addition to NASH and AFLD, viral hepatitis, such as hepatitis B virus (HBV), Hepatitis C virus, can also lead to liver cancer. Autophagosomes (APs) can directly merge with lysosomes (Lys) and degrade and recycle their cargo to maintain cellular homeostasis. Tunicamycin (TM) I, an N-glycosylation inhibitor and ER stress inducer, can trigger ER stress, which enhances autophagy. Experimental results showed that HBV infection induced ER stress and early autophagy, but inhibited autophagic degradation in liver tissue (66). Treating HCC cells with TM revealed that N-glycosylation was not critical for hepatitis B surface antigen (hBsAg) release. However, TM enhanced HBV replication, increased the release of HBV subviral particles (SVPs) and nude capsid, and caused HBV/hBsAg/SVPs to accumulate in the ER under ER stress. In turn, these effects promoted virus production and release. TM also partially reduced the association of hBsAg/HBV/SVPs with early endosomes and significantly increased the recruitment of APs. Further, TM blocked AP-Lys fusion, leading to the formation of exosomes after AP fusion with late endosomes (LEs) to increase cargo (hBsAg/SVPs/nude capsid) output. AP, as an alternative pathway for HBV assembly and release, promoted HBV replication and release of SVPs and nude capsid through the AP-LE/MVB axis under TM-induced ER stress. Although HBV infection can induce autophagy early, it is questionable whether autophagy is inhibited after persistent infection, as it has been found in HCV. Chronic HCV infection leads to an increased accumulation of misfolded proteins in hepatocytes, and when the stress on these proteins cannot be relieved, it causes ER stress, making infected cells susceptible to additional stress, and this increased stress during persistent infection impairs autophagic degradation of infected cells, leading to reduced clearance of misfolded proteins and cellular components, which ultimately promotes the release of EVs, leading to persistent viral infection, causing the development of liver fibrosis and HCC (28). This also supports the fact that there is often a better prognosis in the early stages of the disease

In conclusion, ER stress promotes the release of EVs rich in PD-L1, which not only affects OSCC, BC, but also HCC. As seen in other diseases, ER stress promotes the release of EVs from HCC cells, and miRNA-23a-3p in EVs is transferred to Mφs, which then polarize to the M2 phenotype. At first, the expression of inflammatory factors IL-6, TNF-α and IL-10 is increased, suggesting that HCC cells may induce Mφs to trend towards tumor-associated macrophages (TAMs), thus promoting disease progression. Additionally, PTEN is specifically targeted for inhibition, which activates the PI3K/AKT pathway, upregulates PD-L1 expression, and increases apoptosis of T cells, thus promoting tumor immune escape (67). The same pathway in BC is activated in HCC through different miRNAs, causing the same outcomes. This effect demonstrates the diversity and unity of disease development due to interactions between ER stress and EVs, outside the ER stress-EV-Mφs pathway. These interactions need to be verified in more diseases to provide the basis for disease treatment, especially for diseases of malignancy. In addition to PD-L1, EVs transported to Mφs can promote the expression of cytokines IL-6, IL-10, and MCP-1 by activating the Janus kinase 2-signal transducer and activator of transcription 3 (JAK2/STAT3) pathway, leading to immunosuppression of Mφs and promotion of tumor development (68). These similar mechanisms of action may lead to different outcomes because of similar or dissimilar EV contents and activation pathways, suggesting both opportunities and challenges for disease treatment. ER stress mainly interferes with the proteome in EVs, which in turn orchestrates lipid dynamics in the liver. However, the initial role of ER-stress induced EVs in excreting waste products and mitigating liver disease by directly carrying unmetabolizable lipids out of the liver should be investigated as a potential path for therapeutic research.

## Metabolism-related diseases

Diabetes mellitus, a common metabolic disease, often leads to serious complications without intervention. Although islet transplantation is an effective treatment for severe type 1 diabetes (known as brittle diabetes), some patients develop islet destruction or dysfunction secondary to hypoxia after transplantation, which affects treatment outcomes. Hypoxic conditions were shown to increase β-cell apoptosis through induction of ER stress and p38/MAPK activation, which was the molecular mechanism responsible for poor islet transplantation outcomes (30). Treatment with miRNA-21-enriched EVs derived from mesenchymal stem cells (MSC-EVs) inhibited ER stress and phosphorylation of proteins in the p38/MAPK signaling pathway, apoptosis-related proteins, and caspase 3, while increasing the expression of the anti-apoptotic protein, survivin. These combined effects significantly increased β-cell viability, inhibited β-cell apoptosis under hypoxic conditions, and promoted islet survival and function. These results suggest that MSC-EV treatment may reduce hypoxia after islet transplantation.

In addition to their therapeutic relationship with diabetes, ER stress and EV interactions also affect the pathogenesis of diabetes. Type 1 diabetes is an autoimmune disease. Under pro-inflammatory cytokine-induced ER stress, EVs released by pancreatic β-cells, called immunogenic EVs, were shown to be enriched with autoantigens GAD65, IA-2, and insulin/insulinogen. These autoantigens triggered autoimmunity and the release of immunostimulatory ER chaperones that were taken up by antigen-presenting cells and presented to self-reactive T-cells, thus promoting antigen presentation, T-cell activation, B-cell autoimmunity, and disease development (43). Understanding this pathway may provide inspiration for the treatment of type I diabetes. Various complications caused by diabetes comprise the worst aspects of the disease. For example, diabetic peripheral neuropathy (DPN) is a common complication of diabetes. Apoptosis induced by ER stress in dorsal root ganglion neurons (DRGns), the target cells of DPN injury, is an important mechanism of DPN pathogenesis. High glucose levels induce ER stress, but treatment with paeoniflorin (PF) reduced ER stress by inhibiting GRP78 expression and achieved anti-inflammatory effects by suppressing the phosphorylation of IRE1α (44). Schwann cell (SC)-derived EVs (SC-EVs) carried ER stress initiators and IRE1α pathway initiators to signal to DRGn cells in a hyperosmotic environment that did not affect the activity of SCs and SC-EVs, thus leading to disease progression. However, this effect was exacerbated under high glucose conditions. PF-treated SC-EVs modulated the expression of IRE1α and GRP78, key proteins in the IRE1α signaling pathway during ER stress, in DRGns under high glucose conditions. These effects improved the disrupted morphology of the ER in DRGns, increased the expression of anti-apoptotic protein Bcl-2, decreased the expression of pro-apoptotic protein Bax, and inhibited the expression of caspases 12 and 3. Ultimately, apoptosis was reduced in DRGns and DPN was improved, suggesting a new approach for the treatment of DPN at the molecular level.

Sometimes diabetes is more easily detected than diseases that can lead to diabetes. For example, the loss of glycemic control in pancreatic-derived diabetes (also known as type 3C diabetes mellitus, T3cDM) is often the precursor of chronic pancreatitis (CP) or even pancreatic ductal adenocarcinoma (PDAC). Adrenomedullin (AM) is a hypotensive hormone with proliferative and pro-angiogenic effects that can also inhibit insulin secretion. Pancreatic cancer cells have been shown to preferentially release EVs in the portal and peripheral venous blood of patients with PDAC (45, 69). These EVs carried AM and were internalized by β-cells through microfilament cellular drinking and niche protein-mediated endocytosis, thus inducing increased BIP and CHOP mRNA expression and enhancing ER stress. Further, EVs enhanced AM receptor interactions and upregulated and overstimulated cAMP-dependent pathways, while enhancing apoptosis and BIP-insulinogen interactions, leading to the failure of AM-induced UPR and inhibiting insulin secretion from pancreatic islet cells (45, 69). The addition of AM inhibitory peptide reduced these interactions, which explained the association between PDAC-associated T3cDM (called PC-DM), insulin resistance, and elevated peripheral insulin levels, whereas T3cDM in CP was associated with reduced insulin levels due to insulin-secreting β-cell dysfunction. More research is needed to determine whether EVs can be used in a sensitive and specific screening test for early diagnosis of PDAC.

Equine metabolic syndrome (EMS) is a specific metabolic disorder in horses characterized by insulin resistance, previous chronic laminitis, and obesity in specific areas (e.g., around the eyes or the base of the tail). One study reported that 5-azacitidine and resveratrol (AZA/RES) treatment of adipose-derived MSCs (ASCs) produced EVs with unique biological characteristics (AZA/RES-EVs) (70). Subsequent translocation of AZA/RES-EVs to ASCs isolated from animals with EMS resulted in significantly upregulated anti-apoptotic Bcl-2, downregulated apoptotic Bax, and reduced expression levels of ATF-6, IRE-1, PERK, EIF2, and CHOP in recipient cells. These effects indicated that AZA/RES-EVs not only inhibited apoptosis and reduced ER stress, but also improved cell viability. In short, AZA/RES-treated ASCs promoted the secretion of EVs in recipient cells, which reduced oxidative stress, inflammation, and ER stress, thus protecting cells from apoptosis and senescence. This finding suggests that AZA/RES may provide a therapeutic measure for EMS, but more clinical studies are needed.

Diabetes and EMS are cardiovascular risk factors, and patients/animals may develop endothelial dysfunction without intervention. This leads to an imbalance in vascular homeostasis and often to the development cardiovascular complications, such as early onset of atherosclerotic pulses. ER stress is thought to be a powerful molecular link between insulin resistance, inflammation, and endothelial dysfunction. ER stress can lead to an increase in EV abundance in endothelial cells, and ER stress-induced EVs can, in turn, activate ER stress in healthy endothelial cells. This combination of events creates a vicious cycle between activation of ER stress and production of EVs. Interestingly, although EVs produced by normal endothelial cells and ER-stressed endothelial cells do not differ quantitatively, they differ qualitatively. EVs released during ER stress communicate detrimental biological information about endothelial function, such as angiogenic capacity and increased release of inflammatory factor IL-6, without affecting apoptosis, autophagy, or antioxidant production. Therefore, some complications can be prevented by controlling the release of EVs, inhibiting their uptake by receptor cells, or regulating their cargo content (71). In addition to EVs from endothelial cells, circulating EVs (C-EVs) and EVs from apoptotic T cells are also capable of inducing endothelial dysfunction. In patients with metabolic syndrome, apoptotic T cells act on Fas and low-density lipoprotein receptor (LDL-R) to activate ER stress (72). Through interactions between the ER and mitochondria, mitochondrial ROS levels increase, leading to decreased NO bioavailability, impaired endothelium-dependent vasodilatory function, and ultimately endothelial dysfunction. In contrast, C-EVs act on Fas/FasL to direct endothelial dysfunction, but not LDL-R. Thus, the mechanisms associated with the induction of ER stress differ slightly between the two types of EVs, which may be due to differences in their sources.

## Neurological diseases

Neurodegeneration, due to protein misfolding and aggregation, occurs in a variety of brain disorders, such as Parkinson’s disease, Alzheimer’s disease (AD), and Huntington’s disease (HD), collectively known as protein misfolding disorders. HD is an autosomal disorder caused by mutations in the huntingtin protein (Htt) gene, which results in the accumulation of intracellular mutant Htt (mHtt) and polyglutamine peptide aggregates. The protein deposition network is an important way for cells to maintain protein homeostasis and relies heavily on the ER to prevent abnormal protein accumulation. However, this ability becomes diminished with aging, leading to ER stress, protein misfolding and accumulation within the cell, and eventually disease. Gene disruption in the IRE1α/XBP1 pathway was found to lead to HD (73). XBP1 conditional knockout mice, as an animal model of HD, were used to determine that insulin-like growth factor 2 (IGF2) upregulation may act as a backup mechanism for maintaining neuronal function. Additionally, cell culture models of HD showed that IGF2 treatment enhanced the unconventional disposal of soluble polyglutamine peptide material into the extracellular space through secretion of EVs, thus reducing the accumulation of intracellular mHtt and polyglutamine peptide aggregates. IGF2 not only controlled protein deposition and mHtt aggregation, but also affected neurons and synapses to ameliorate disease symptoms. In summary, the protein deposition network becomes less functional during aging and ER stress and protein accumulation triggers disease, whereas IGF2 reduces the misfolded protein load and slows down disease development through extracellular disposal by EVs without affecting two major degradation routes, proteasomes and macroautophagy.

In AD, ER stress and EVs have complex mechanisms of action on disease progression. AD pathology is characterized by granulovacuolar degeneration (GVD) and hyperphosphorylated tau (pTau). Compared with normal brains, brains with AD lack ER chaperone and binding proteins in submacular neurons, and abnormal protein homeostasis causes neuronal ER stress. pTau aggregates also form neurogenic fiber tangles with abnormal cytoplasmic RNA binding protein (RBP) aggregates such as matrin 3, fused in sarcoma, and stress granules. Furthermore, impaired autophagy is regulated by both the ER and RBPs. All the above effects eventually lead to the accumulation of GVD bodies (GVBs), thus causing GVD. In addition, GVBs consistently co-localize with the EV marker flotillin 1, suggesting that the transfer of GVBs *via* EVs may underlie the transneuronal spread of neurodegenerative lesions (74). Together, these results are compatible with the notion that GVBs might be exocytosed just like EVs. However, this hypothesis must be validated by functional/experimental studies. ER stress leads to release of EVs and transfer of their contents, resulting in disease progression. Therefore, interrupting this pathway could reduce ER stress and prevent further disease progression.

The rate of degradation of cyclic adenosine monophosphate (cAMP) is regulated by phosphodiesterases, of which PDE4A is a member. Phosphodiesterases are enhancers thought to be a potential treatment for AD. In one study, circular AXL receptor tyrosine kinase (circAXL) derived from serum-EVs was found to be upregulated in patients with AD, whereas the target miR-1306-5p was downregulated (31). Subsequently, an AD cell model was constructed by treating SK-N-SH neuronal cells with Aβ1-42, which is known to trigger a series of neuronal injuries such as neuronal cytotoxicity, apoptosis, inflammation, and oxidative stress. Treated SK-N-SH cells displayed significantly enhanced PDE4A expression and decreased levels of cAMP. In contrast, downregulation of circAXL relieved the inhibition of miR-1306-5p, thus reducing the expression of PDE4A and largely alleviating the damage caused by Aβ1-42. This finding suggests that circAXL may regulate the expression of PDE4A by targeting miR-1306-5p, thereby affecting cAMP concentrations. In brief, circAXL is involved in Aβ1-42-induced neuronal injury by targeting the miR-1306-5p/PDE4A axis. Therefore, circAXL and miR-1306-5p in EVs can be used not only as diagnostic markers for AD, but inhibition of circAXL or enhancement of miR-1306-5p can attenuate Aβ1-42-induced neurotoxicity in AD, suggesting paths for future research.

One study examining the underlying molecular mechanisms of the strongest common genetic risk factor for PD, the glucocerebrosidase gene (GBA) encoding the lysosomal enzyme GCase, reported that the heterozygous GBA-N370S mutation caused dopaminergic neuronal malfunction as evidenced by the retention of misfolded GCase proteins in the ER, disrupted dopaminergic neuronal proteins, ER stress, autophagy/lysosome dysfunction, and increased α-synuclein release, eventually leading to preferential dopamine neuronal vulnerability in PD (75).

Remote ischemic post-conditioning (RIPostC), a technique that protects vital organs in an indirect manner through remote EV-mediated transfer of functional factors, is being used to treat central nervous system (CNS) diseases. In one study, HUVECs were treated with H/R and their EVs were collected (32). After co-culture with H/R HUVEC-derived EVs, miR-199a-5p was transferred to neuronal cells, resulting in increased miR-199a-5p expression and decreased expression levels of XBP-1, p-PERK, p-eIF2α, ATF4, CHOP, and ATF6. These results indicated that miR-199a-5p inhibited BIP as well as apoptosis and inflammation associated with ER stress. After transfecting neuronal cells with miR199a-5p inhibitor and co-culturing with H/R HUVEC-derived EVs, the anti-H/R function was largely reduced. The study findings indicated that the long-distance effect of H/R HUVEC-derived EVs depended on miR-199a-5p levels in neurons. This study elucidated the mechanism underlying the long-distance protective effect of ischemically injured tissues on the CNS, which might be the mechanism responsible for the protective effects of RIPostC against CNS. However, more studies are needed to verify this conclusion. In another study, LPS-treated PC12 cells were employed to construct a spinal cord injury (SCI) model, revealing that miR-9-5p expression in EVs derived from bone marrow MSCs (BMSCs) promoted the upregulation of fibroblast growth factor 2 (FGF2) expression through targeting histone deacetylase 5 (HDAC5)-mediated deacetylation, thereby inhibiting LPS-induced apoptosis, inflammation, and ER stress (33). HDAC5 overexpression or FGF2 inhibition reversed the effects of miR-9-5p in EVs and attenuated inflammation and ER stress in SCI rats after treatment with BMSC-EVs. Further research is warranted to determine if different types of neurological disorders caused by common causes can be treated using the same approach. For example, neurological disorders caused by protein misfolding and aggregation may be alleviated by the uniform reduction of these proteins.

## Orthopedic diseases

For orthopedic diseases, the ER and EVs have been mostly studied in relation to the treatment of intervertebral disk (IVD) degeneration (IVDD) and steroid-induced osteonecrosis of the femoral head (ONFH). An IVD consists of an internal nucleus pulposus (NP), an external fibrous ring (annulus fibrosus, AF), and an ascending cartilaginous endplate (CEP). Concerning these three components, studies have mostly targeted the AF and CEP, but inhibiting apoptosis of NP cells during IVDD may be an effective way to prevent or reverse disk degeneration. Treatment with advanced glycation end products (AGEs) was shown to promote CHOP expression and lead to increased apoptosis of human NP cells (34). However, when AKT or ERK signaling was blocked, the expression of both CHOP and cleaved caspase-3 was decreased. These findings indicated that treatment of NP cells with AGEs induced severe ER stress, leading to the accumulation of CHOP protein and dephosphorylation of AKT and ERK, which promoted cleavage of caspases 12 and 3 and resulted in apoptosis. Inhibition of AGE-induced ER stress and restoration of apoptotic, damaged, or aged NP cells may provide an approach for treating IVDD. In another study, EVs derived from BMSCs were found to attenuate apoptosis in human NP cells (35). Administration of BMSC-EVs restored impaired AKT and ERK signaling, attenuated ER stress induced by AGEs, reduced CHOP expression, and ultimately protected NP cells from excessive apoptosis. In contrast, the anti-apoptotic effect of BMSC-EVs was significantly diminished when treated with PI3K/AKT or ERK inhibitors. This finding suggests that BMSC-EVs ameliorated ER stress-induced apoptosis in part by activating AKT and ERK signaling pathways. The IVD is the largest avascular structure in the body and receives all of its nutrients from the bone marrow of adjacent vertebrae. Apoptosis and calcification of endplate chondrocytes (EPCs) interfere with endplate nutrient transport, leading to NP degeneration and exacerbation of IVDD. Not only can BMSC-EVs protect NP cells, they can also slow down disease progression by protecting EPCs from calcification and apoptosis. Under tert-butyl hydroperoxide-induced oxidative stress, BMSC-EVs delivered to EPCs resulted in increased miR-31-5p expression and decreased expression levels of ATF6, CHOP, XBP1, and GRP78, thus disrupting ATF6-related ER stress and regulating cellular function. These results indicated that BMSC-EVs protected EPCs from oxidative stress-induced apoptosis, but also inhibited oxidative stress-induced calcification. Briefly, BMSC-EVs protect EPCs from apoptosis and calcification through the miR-31-5p/ATF6-related ER-stress pathway, suggesting that BMSC-EVs can contribute to the treatment of IVDD by targeting the NP and CEP.

As previously mentioned, EVs are present in almost all biological fluids, including urine, and some studies have demonstrated that human urine-derived stem cell EVs (USC-EVs) can have therapeutic effects. Excessive mechanical stress was shown to lead to ER stress, causing apoptosis of NP cells and ultimately IVDD (46). NP cells cultured under pressurized gas exhibited increased expression levels of GRP78, CHOP, and caspases 12 and 3, with increasing pressurization time, which suggested that ER stress was induced under mechanical stress. Co-culture with USC-EVs reduced expression levels of CHOP and caspases 3 and 12, which promoted phosphorylation of AKT and ERK, activated AKT and ERK signaling pathways, and inhibited ER stress-induced apoptosis of NP cells. The anti-apoptotic effect of USC-EVs was attenuated when cells were treated with AKT or ERK antagonists. Briefly, USC-EVs significantly improved the ER stress response and inhibited overactivation of the UPR, as well as apoptosis and disk degeneration, through AKT and ERK signaling pathways. Taken together, these results suggest that either BMSC-EVs or USC-EVs may be used as a potential strategy for the treatment of IVDD.

In addition to IVDD, interactions between ER stress and EVs can be observed in ONFH. Widespread use of glucocorticoids has resulted in ONFH becoming increasingly common, owing to cell death caused by high doses of glucocorticoids. Inhibition of endothelial cell and osteoblast apoptosis under ER stress has been suggested as a potential target for the treatment of ONFH. Platelet-rich plasma (PRP) contains many growth factors. EVs derived from PRP (PRP-EVs) were found to have similar effects as their parent bodies and were rich in growth factors. In endothelial cells, PRP-EVs were shown to enhance AKT activation under ER stress (47). Subsequently, activated AKT inactivated Bad (Bcl-2-related death promoter), which promoted cell death, increased the expression of Bcl-2, and prevented activation of caspase-3. Moreover, PRP-EVs are enriched with vascular endothelial growth factor (VEGF), a key factor for angiogenesis. VEGF-A binding to phosphorylated VEGFR2 led to MAPK/Erk1/2 activation, which promoted proliferation, survival, and migration of endothelial cells, thus promoting angiogenesis. In osteoblasts, PRP-EVs enhanced the activation of AKT, which triggered phosphorylation-mediated inactivation of glycogen synthase 3β (GSK-3β), causing the accumulation and stabilization of β-catenin, which ultimately maintained the osteogenic capacity of BMSCs and other preosteoblasts. These findings indicated that PRP-EVs can act on two types of cells (endothelial cells and osteoblasts) simultaneously to slow down disease progression, on the one hand promoting angiogenesis, and on the other hand maintaining the osteogenic capacity of osteoblasts, which may provide inspiration for other studies.

## Other diseases

Interactions between ER stress and EVs can affect diseases other than those mentioned above. Endothelial corneal dystrophy caused primarily by ER stress is a common cause of corneal vision loss. Using an *in vitro* model of corneal endothelial injury induced by serum deprivation, one study reported that MSC-EVs activated the AKT pathway and inhibited eIF2, a counter-regulator of AKT (48). The restoration of AKT phosphorylation levels led to a significant downregulation of pro-apoptotic caspase-3 expression and significantly reduced apoptosis of human corneal endothelial cells (HCECs), demonstrating the pro-angiogenic effect of MSC-EVs. Although EVs derived from healthy donor sera (SER-EVs) could restore AKT levels affected by ER stress, SER-EVs had a much lower regulatory effect on ER stress-related genes than MSC-EVs, and did not decrease caspase-3 levels or reduce the number of apoptotic HCECs. These findings suggest a potential therapeutic effect of MSC-EVs, but not SER-EVs, on ER stress activation in corneal endothelial lesions, perhaps by direct intra-atrial injection of MSC-EVs. In another study, BMSC-EVs protected the kidney against renal I/R injury (36). ER stress was significantly induced after renal I/R, and the supernatant of BMSCs (without foreign bodies) reduced I/R-induced apoptosis. However, their effect was less pronounced than that of BMSC-EVs, which peaked in the very early reperfusion phase, a period when ER stress was significantly induced and a critical period for apoptosis and cell death. These results suggest a time-dependent effect of BMSC-EVs. In addition, BMSC-EVs were rich in miR-199a-5p, and the miR-199a-5p content in renal tubular epithelial cells (NRK-52E) increased significantly after EV transfer, indicating that miR-199a-5p could inhibit ER stress by targeting BIP. Although the effects of BMSC-EVs and miRNAs were not transient, the inhibitory effects were more significant in the early stage of reperfusion, slowing down cell death and apoptosis to effectively protect the kidney.

However, interactions between ER stress and EVs do not play a role in all diseases. For example, induced abortion mostly because of chromosomal abnormalities can be triggered by factors such as advanced maternal age and smoking. Studies have shown that the accumulation of senescent cells during pregnancy can lead to pregnancy complications. In one study, oxidative stress was shown to induce ER stress in placentas from missed miscarriages, leading to accumulation of misfolded proteins in the ER that became toxic and harmful to cell survival (76, 77). However, levels of misfolded proteins were not increased in EVs of placentas from missed miscarriages, indicating that these potentially dangerous proteins were not exported to EVs and that increased ER stress in the placenta was not released through EVs. This finding suggests that ER stress in the placenta, but not transmitted through EVs, may be involved in the pathogenesis of missed miscarriage. In contrast, another study reported that antiphospholipid autoantibodies, risk factors for preeclampsia and miscarriage, induced ER stress in the syncytial trophectoderm (76, 77). The syncytial trophectoderm exported potentially dangerous proteins in EVs, including misfolded proteins and those of mixed lineage kinase domains like pseudokinase, to avoid their accumulation and toxicity. The removal of misfolded proteins by EVs protected the stressed syncytial trophectoderm, but these proteins were potentially toxic to maternal cells releasing placental EVs and may be a contributing factor to endothelial cell activation, a marker of pre-eclampsia or maternal organ damage. Although the appearance of opposing functions of placental EVs in these two studies may be due to differences in the placental microenvironment, these studies provide evidence that EVs can transmit ER stress. Nevertheless, ER stress is not negatively regulated in all diseases. *Lactobacillus paracasei* (Lp) isolated from the vagina can have anti-inflammatory effects, such as reducing the severity of inflammatory bowel disease symptoms by regulating the expression of cytokines, inflammatory mediators, and NO production (78). Indeed, Lp-derived EVs (Lp-EVs) were shown to inhibit LPS-mediated intestinal inflammation by inducing ER stress. These results suggest that Lp-EVs could be used not only for the treatment of gastrointestinal disorders, but also for the treatment of vaginal ecological disorders, although more evidence is needed to support their clinical use.

## Conclusion

**Figure 2:**
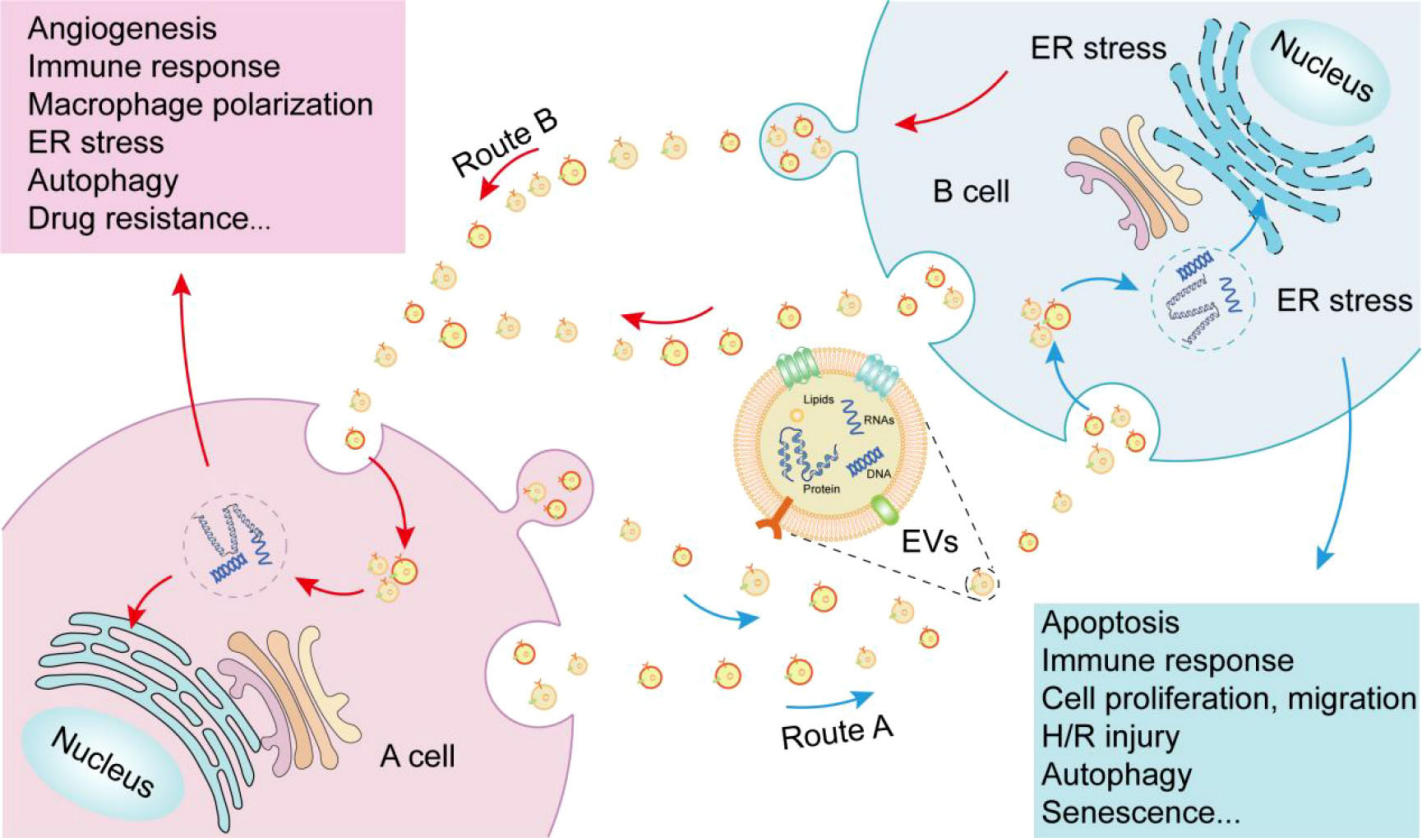
Interactions between endoplasmic reticulum stress (ER) and extracellular vesicles (EVs). Route A: EVs released by A cell are internalized by B cell, and the contents of EVs cause ER stress in B cell, ultimately causing a number of outcomes in B cell. Route B: EVs released by B cell under ER stress are internalized by A cell, which can transmit endoplasmic reticulum stress to A cell and cause a series of outcomes in A cell.

EVs are both a cause and consequence of ER stress. Depending on the magnitude of ER stress, abundance of EVs, cell type, and specific pathological context, interactions between ER stress and EVs can affects multiple disease processes, such as tumor growth, cell proliferation, angiogenesis, immune responses, and apoptosis (**Figure 2**). Although a multitude of diseases are caused by protein accumulation due to ER stress, many of the interactions between ER stress and EVs remain to be elucidated. Indeed, the complex mechanisms of disease onset prevent the singular assumption that ER stress leads to EV release and transport, or that EVs lead to ER stress. Rather, ER stress and EVs are involved in a complex cyclic process and the specific mechanism by which many EVs act remains unclear. Blocking parts of this cyclic process as therapeutic targets for certain diseases provides direction for future research. In addition, the application of EVs in bioengineering to alleviate ER stress remains to be explored. Identifying additional signaling convergence points will guide the development of powerful therapeutics for autoimmune diseases and other UPR-mediated disorders. Interactions between ER stress and EVs will likely play an important role in various aspects of medical treatment in the future.

## Author contributions

JY wrote the manuscript. XL conceived the study. All authors have reviewed and approved the final version of the manuscript.

## Acknowledgments

We would like to thank Editage (www.editage.cn) for English language editing.

## Conflict of interest

The authors declare that the research was conducted in the absence of any commercial or financial relationships that could be construed as a potential conflict of interest.

## Publisher’s note

All claims expressed in this article are solely those of the authors and do not necessarily represent those of their affiliated organizations, or those of the publisher, the editors and the reviewers. Any product that may be evaluated in this article, or claim that may be made by its manufacturer, is not guaranteed or endorsed by the publisher.
